# Early thrombus detection in ECMO with optimized impedance measurements: A simulative study

**DOI:** 10.2478/joeb-2025-0011

**Published:** 2025-07-01

**Authors:** Filip Slapal, Diogo F. Silva, Steffen Leonhardt, Marian Walter

**Affiliations:** Medical Information Technology, RWTH Aachen University, Germany; Department of Circuit Theory, CTU Prague, Czechia

**Keywords:** thrombus detection, finite element method, machine learning, sensitivity analysis

## Abstract

Extracorporeal oxygenation supports patients with severe cardiac or respiratory failure, with the oxygenator providing critical gas exchange. Thrombus formation in the oxygenator can impair efficiency and increase risks such as hemolysis and embolism, but existing detection methods are limited in accuracy and timeliness. This study introduces a computational bioimpedance approach for early thrombus detection that integrates advanced modeling and machine learning techniques while preserving the oxygenator’s functionality.

We developed a finite element model of an oxygenator to simulate bioimpedance measurements using varied electrode configurations. Neural networks optimized electrode placement and injection-measurement patterns, enhancing sensitivity to conductivity changes. A second neural network was trained on simulated data to distinguish between normal and thrombus-affected conditions, achieving an F1-score exceeding 94% in classification tasks.

Simulations demonstrated the feasibility of this method, with optimized configurations significantly improving detection accuracy. The findings suggest that computational bioimpedance, combined with neural network optimization, provides a robust framework for automated thrombus detection inside an oxygenator.

## Introduction

### ECMO oxygenators

Extracorporeal membrane oxygenation (ECMO) is a life-sustaining intervention used to support patients with severe cardiac or respiratory failure who require temporary extracorporeal gas exchange. This advanced life support technique is often employed when conventional treatment methods, such as mechanical ventilation or pharmacological therapies, are insufficient to provide adequate oxygenation and carbon dioxide removal. ECMO serves as a critical bridge to recovery, transplantation, or decision-making regarding long-term therapeutic strategies [1].

ECMO has a wide range of applications across various patient populations, including neonates, children, and adults. In neonatal intensive care, ECMO is utilized for conditions such as persistent pulmonary hypertension of the newborn, meconium aspiration syndrome, congenital diaphragmatic hernia, and severe respiratory distress that is unresponsive to conventional treatments. In pediatric and adult patients, indications for ECMO include severe cases of acute respiratory distress syndrome (ARDS), refractory hypoxemia, and respiratory failure due to viral or bacterial pneumonia, including infections such as influenza and COVID-19.

A fundamental constituent of the extracorporeal circuit is the oxygenator, which plays a pivotal role in the process. Current typical designs incorporate an assembly of hollow fibers, constructed from gas-permeable material, arranged in bundles. These fibers facilitate the critical exchange of oxygen and carbon dioxide, thereby enabling effective gas transfer within the circuit.

Despite its remarkable benefits, ECMO is associated with various challenges and complications, including haemorrhage, thrombosis and infection [2]. With up to 53% of the cases, thrombosis is a relatively common occurrence during ECMO treatment [3], which can manifest in several parts of the ECMO circuit or the patient’s circulation.

### Blood clots in oxygenators

Unfortunately, the details of oxygenator thrombus formation are not well understood. However, it has been documented that fibrous or cellular debris deposited on membrane fibers may not only result in decreased gas exchange capabilities, but also increase blood flow resistance and the risk of hemolysis, further activation of coagulation, or, if dislodged, thromboembolic complications [4].

Various parameters are being monitored in clinics in order to detect clots during ECMO treatment. Three main methods are regularly used for assessing the fitness of the oxygenator [5]:

- Visual inspection of the externally visible parts of the oxygenator and tubing is the simplest method. However, it does not reveal thrombi hidden within the oxygenator.
- Measurement of partial oxygen (O_2_) and carbon dioxide (CO_2_) pressures in the oxygenator outlet are directly influenced by thrombus formation on the membrane surface and thus reveal possible thrombi on the membrane surface.
- Measurement of a pressure drop on the oxygenator between one pressure sensor placed upstream and a second sensor downstream of the oxygenator can point to thrombi presence through an increased flow resistance.

In summary, the aforementioned methodologies tend to increase the workload of personnel and may only result in a simplified global analysis of thrombi presence, missing earlier or hidden stages of thrombi formation.

### Bioimpedance-based thrombi detection

In order to prevent acute ECMO complications related to thrombus formation, which may necessitate immediate oxygenator replacement, an automated and dependable method for early blood clot detection would be highly advantageous. One potential approach to this could be distinguishing blood from blood clots based on their bioimpedance. We note that the difference in bioimpedance between blood and thrombus has been demonstrated in several studies in the past. Already in 1930, Gelfan and Quigley [6] showed that thrombus impedance starts to rise sharply approximately ten minutes after the start of coagulation. Noshiro et al. [7] observed in 2007 a conductivity difference around 4 mS·cm^−1^ = 0.4 S·m^−1^ between normal and coagulated blood in a frequency range from 100 kHz to 800 kHz. This difference decreases with increasing frequencies above 800 kHz.

Although this principle of blood clot detection has been utilised by several researchers, for example in 2015 by Sapkota et al. [8], in 2021 by Chen et al. [9] or in 2023 by Türkmen et al. [5], there are some factors complicating its use. Blood impedance is dependent not only on the blood coagulation state, but also on the hematocrit value, flow, erythrocyte orientation or temperature. The impedance of the blood clot alone is also influenced by clot composition adding more variables to consider for method development [5, 10, 11].

The goal of this article is to design an autonomous and reliable method of thrombus detection based on multielectrode bioimpedance measurements with detection electrodes placed on the oxygenator surface in a way not to interfere with oxygenator normal function. We developed a simulation framework for bioimpedance measurements with an electrode array based on a simplified finite element method (FEM) model of a real oxygenator. By coupling neural networks (NNs) to this environment, we then optimized the electrode positions and measurement patterns for maximal sensitivity, and trained a classifier for thrombi detection.

## Materials and methods

### FEM modelling

#### Oxygenator FEM model

The MATLAB-based open-source framework EIDORS (Polydorides, 2002) was utilised for all simulations due to its user-friendly finite element modelling interface for the analysis of multi-electrode impedance measurement systems.

**Figure 1:**
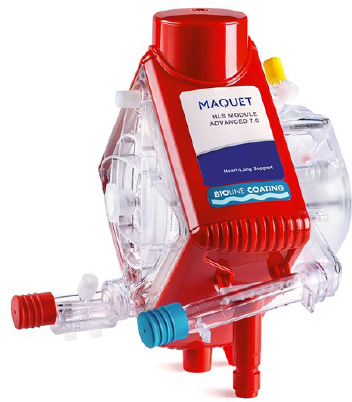
’HLS Module Advanced’ oxygenator by Maquet Cardiopulmonary GmbH (Rastatt, Germany).

**Figure 2:**
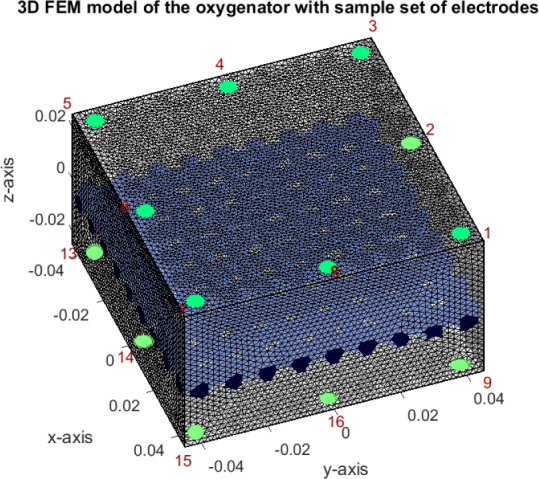
The generated FEM model of the oxygenator with a sample electrode array (depicted in green) and separation grid (depicted in blue). Red is used to number visualized electrodes.

A simplified model of the oxygenator from Maquet Cardiopulmonary GmbH (Rastatt, Germany), the ’HLS Module Advanced’ (see Figure 1), was chosen for this study. Its discretised finite FEM model containing tetrahedral elements was created for the simulations. It shares the same inner-volume dimensions with the oxygenator. Additionally, a grid of available electrode positions was prepared to facilitate contact with blood. A symmetrical grid was placed only at top and bottom planes because the construction of the actual oxygenator does not allow for other placement. The actual electrode positions were then chosen from these available options. A separation grid was created in the middle of the model to emulate a compartment divider present in the oxygenator. In the actual module, the separation grid is a polycarbonate lattice running orthogonally through the fiber bundle, ensuring proper fiber alignment and uniform blood flow distribution. For the FEM model, we approximated the manufacturer’s geometry as faithfully as possible using meshed cylindrical rods, and reflected its essentially non-conductive nature by setting the electrical conductivity of the grid to 1 × 10^−6^ S·m^−1^, as per Table (1). For simulation feasibility, individual oxygenator fibers were not modeled. The maximal mesh-size was set to 2 mm as a compromise of good simulation accuracy and feasible memory requirements. The final generated mesh (depicted in Figure 2) includes 373,202 elements and 70,914 nodes.

**Table 1:** Overview of the FEM model component properties.

Part	Conductivity	Characteristics
BG	6.62 × 10^−1^ S·m^−1^	9 × 9 × 5 cm
Clot	6.62 × 10^−2^ S·m^−1^	Spherical targets
SG	1 ×10^−6^ S·m^−1^	Rod diameter 0.4 cm

The conductivity of blood in this work was set at 6.62 × 10^−1^ S·m^−1^ according to the IT’IS Foundation database [12]. The conductivity of targets emulating clots was chosen to be approximately one-tenth of blood conductivity (6.62 × 10^−2^ S·m^−1^) according to [7]. It is hypothesised that the discrepancy in conductivity between blood and thrombi is likely to exceed the mean conductivity difference in vivo, thereby representing the optimal scenario for thrombus detection.

Individual component properties are specified in Table 1 with BG corresponding to background and SG to separation grid.

#### Measurement simulations

The potentials on the oxygenator boundaries are calculated by solving a so-called ’forward problem (FP)’ based on the known electric current and known conductivity distribution inside the oxygenator. The FEM volume discretization under the assumption of linear propagation of the current between the FEM elements yields the linear equation (1) for calculating the potentials

(1) v=F(σ,q),

where ***v*** represents the vector of measured voltages at surface electrodes, ***σ*** represents the matrix of conductivity distribution and ***q*** represents the vector of the current injection pattern.

Based on the FP, we can compute a Jacobian sensitivity matrix (***J*** ) for a given injection-measurement (I-M) pattern. This gives us a way to investigate how sensitive each voltage measurement is to small conductivity changes in the FEM mesh and therefore how well our method reacts to these changes. This matrix is defined by equation (2)

(2) $$J_{i,j}=\frac{\partial v_{i}}{\partial \sigma_{j}},$$

where $J\in {\mathbb{R}}^{n_{v}\times n_{M}}$ has dimensions given by the number of voltage measurements (*n_V_* ) and the number of finite elements (*n_M_*), ***J****_i,j_* represents position i,j in the sensitivity matrix, ***v****_i_* represents the i-th voltage measurement, and ***σ****_j_* represents the j-th finite element. All of the measurements of one frame are squeezed into one column and all of the finite elements are squeezed into one row. This way, all of the sensitivity information is contained in a single matrix.

Regions well covered by each voltage measurement are easily identifiable based on the sensitivity matrix. By maximizing sensitivity in specific regions of interest we can tune measurement parameters such as electrode placement [13, 14].

#### Electrode position generation

**Figure 3:**
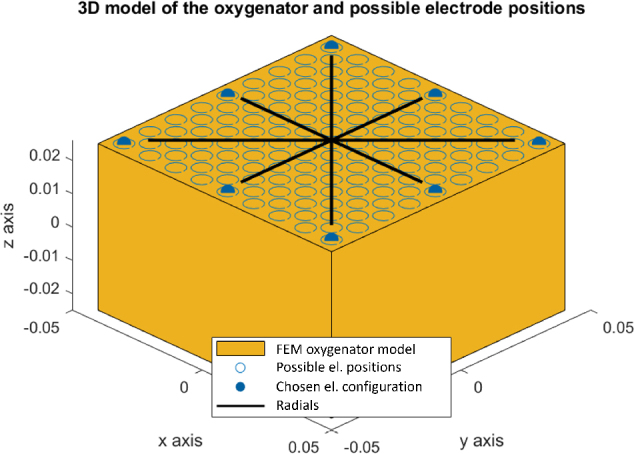
A sample generated electrode array with highlighted radials.

All considered arrangements of electrodes have the same number of electrodes at the top and bottom plane of the model, because there is no evidence suggesting one side of the oxygenator should be disproportionately populated with electrodes. Eight electrodes are therefore generated on the top and eight electrodes at the bottom. Generated electrode centers lie on radials evenly dividing the top and bottom planes (radials are $\frac{360{}^{\circ}}{8}=45{}^{\circ}$ apart). This arrangement promotes rotational invariance with respect to the real module’s geometry, while still reaching corners, where low-flow zones make thrombus deposition most likely. Thrombus formation can also occur unpredictably around the cylindrical axis once localized stagnation develops, hinting at an arrangement that either achieves uniform sensitivity or strategically targets regions near walls. Restricting electrodes to radially arranged slots significantly simplifies the computational complexity: rather than optimizing fully independent three-dimensional coordinates for all electrodes, the algorithm needs only to adjust one radial distance per angular spoke. This simplification considerably reduced computational demands while maintaining the effectiveness of electrode-array optimization. A sample array of electrodes together with highlighted radials is depicted in Figure 3. Both random and supervised position generation was performed on the radials. For the case of random generation, distances from the centre of top or bottom planes are generated from a normal distribution *N* (*µ_elpos_, σ_elpos_*) of a given mean *µ_elpos_* and standard deviation *σ_elpos_* .

#### Target generation

The homogeneous case of the simulations considers only blood and the separation grid in the oxygenator volume. For inhomogeneous simulations, spherical targets are generated. A probability function of thrombi occurrence was derived based on a simplified model of a flow distribution, which assumes a greater flow in the center of the oxygenator and a low flow near the edges and corners of the oxygenator. Recent *in vivo* oxygenator experiments [15] demonstrated that more than four-fifths of thrombi initiate in flow-stagnant rim zones of the fibre bundle, whereas the well-perfused core remains largely clot-free. A 3D Gaussian with mean values *µ_x_* = 0, *µ_y_* = 0 and *µ_z_* = 0 and standard deviations of *σ_x_* = 0.045, *σ_y_* = 0.045 and *σ_z_* = 0.025 was used to empirically approximate the shape of the internal flow field. A probability function of thrombi occurrence was created by analytically inverting the probability density function values for all voxels inside the FEM model according to equation (3)

(3) $$L\left(x,y,z\right)=\sigma_{\chi }\sigma_{y}\sigma_{Z}{\left(2\pi \right)}^{\frac{3}{2}}e^{\frac{{\left(x-\mu x\right)}^{2}}{\sigma_{\chi }^{2}}+\frac{{\left(y-\mu y\right)}^{2}}{\sigma_{y}^{2}}+\frac{{\left(z-\mu z\right)}^{2}}{\sigma_{Z}^{2}}},$$

down-weighting voxels in the high-velocity centre and accentuating those near the walls and corners.

### Measurement optimization

Four-point bioimpedance measurements were chosen to mitigate noise and other effects of nonideal real life conditions. Preliminary simulations revealed that changes in electrode positions and the injection-measurement (I-M) pattern can lead to better signal acquisition. Therefore, an optimization of electrode positions and I-M patterns was performed. This complex problem was decoupled into two separate optimization problems to thoroughly inspect each and simplify the overall optimization.

#### Injection-measurement pattern optimization

**Figure 4:**
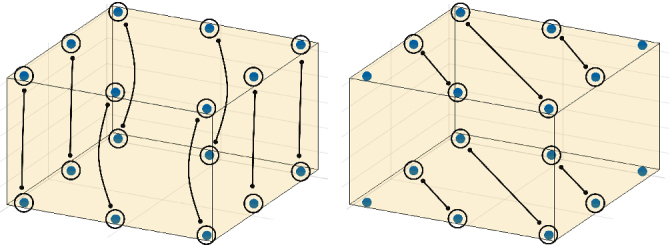
Examples of electrode pairs for inter-plane and intra-plane sensing.

A new I-M pattern was developed to fit the spatial geometry of the oxygenator, with an electrode array placed along the edges of the oxygenator. This was done under the assumption that the optimal electrode configurations will not differ too much from this setup. The array consists of sensible pairs of electrodes chosen in such a way, that their signals include both intraplane and inter-plane excitations and measurements. Selected electrode pairs were used for both injecting and measuring. Only independent measurements in the sense of reciprocity were taken into account. This eliminates the measurement pairs with swapped current injecting and voltage measuring electrode pairs, which do not provide any new information. Examples of the utilized electrode pairs for inter-plane and intra-plane sensing are depicted in Figure 4. Overall, 1560 different measurements were designed. Not all the combinations of injecting and measuring electrode pairs are yielding sensitive measurements and therefore only the most sensitive measurements were selected.

Three methods based on Jacobian analysis were considered for measurement selection. Two of them - the L1- and L2-norms - assess individual measurement sensitivity. These selection methods stem from the idea that the greater the magnitude of the *J* sensitivity vectors (rows with elements corresponding to the given measurement), the better the ability of the measurement to mirror conductivity changes to voltage changes. Vectors with greater magnitude are also more robust to noise. L1-norm and L2-norm were computed according to equations (4) and (5), respectively [14].

(4) $$\|J_{meas}{\|}_{1}=\sum_{k=1}^{n_{M}}\left|J_{meas,k}\right|$$

(5) $$\|J_{meas}{\|}_{2}=\sqrt{\sum_{k=1}^{n_{M}}{\left(J_{meas,k}\right)}^{2}}$$

In this equation, *J_meas,k_* represents a single *J* element corresponding to measurement *meas* and finite element *k*, *n_M_* represents the number of finite elements.

The third method is based on an approach developed by Onsager et al. in 2021 [14] and aims at finding the most sensitive I-M pattern omitting similar measurements. Similar measurements are characterized by their corresponding sensitivity vectors being almost parallel (i.e. they cover the same elements). Independent measurements are, on the other hand, characterized by their corresponding sensitivity vectors being almost orthogonal (they cover different elements). The best I-M patterns chosen based on this selection method include long and mutually orthogonal sensitivity vectors. To reflect this, the sensitivity parallelotope volume formed by the constituent sensitivity vectors was chosen as a metric. A determinant-based computation described in equation (6) was performed

(6) $$V_{n}=\sqrt{\text{ det }\left(J_{meas}J_{meas}^{T}\right)},$$

where matrix *J_meas_* contains only the appropriate *J* sensitivity vectors, which belong to chosen measurements.

#### Electrode position optimization

Electrode positions were optimized with respect to designed configuration figures of merit with a neural surrogate based on Smyl and Liu (2020) [16] which was taught to understand the relationship between selected features representing measurement quality and electrode positions.

Training data for the neural network (NN) was generated using simulations and subsequent feature extraction. Firstly, 8000 random electrode arrays in different spatial arrangements were generated. Variations in the symmetry of electrode placement in one plane and both planes resulted in 2918 unique electrode arrays used for the NN training. An electrode array was considered unique only in case of at least 2 electrodes differing in position from all other electrode arrays.

Only 208 measurements from the designed I-M pattern were kept to correspond with the most common number of measurements used in EIDORS studies. Measurements were selected according to the Table 2.

**Table 2:** Overview of measurement-selection methods and counts.

Maximization	Number of measurements
Parallelotope volume	144
L1-norm	32
L2-norm	32

**Figure 5:**
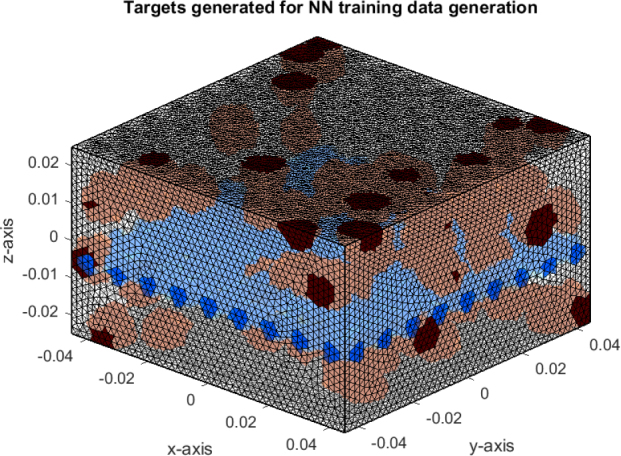
Targets for NN training data generation in red and separation grid in blue.

Twelve sets of six targets each were generated. All six targets within one array were drawn from the thrombus likelihood distribution described in Section ’Target generation’. Targets were generated with percentiles chosen equidistantly spread from 5 to 98 and covering all oxygenator areas. All sets of targets used for training are depicted in Figure 5.

Two main feature groups were designed as inputs for the NN. The *J* was calculated for each electrode array and the relative voltage differences (∆V*_r_* ) between the homogeneous and inhomogeneous cases were calculated from the FP solutions of all combinations of generated target sets and electrode arrays. Singular value decomposition (SVD) as well as L1-norm calculation were performed on the *J* of all 208 sensitivity vectors corresponding to individual measurements. Based on correlation analysis, three figures of merit were selected.

Input data were randomly divided into three parts to form training (80 %), validation (10 %) and test datasets (10 %). A stochastic gradient descent with momentum and a gradually decreasing learning rate of an initial value of 1 × 10^−3^ was selected as a solver. The regularization of NN parameters was included as well as a regularization factor of 1 × 10^−6^. Validation checks were performed regularly to prevent overfitting to the training dataset. Overall, 200 training epochs were completed.

The structure of the NN was adapted from Smyl and Liu [16] with the output being adjusted to accommodate an extra dimension to work in 3D. It consists of two fully-connected (FC) hidden layers followed by a leaky rectified linear unit (ReLU) activation function layers. The number of neurons in layer 1 (*n_l_*_1_) is given by equation (7)

(7) $$n_{/1}=\text{floor}\left(\sqrt{\left(N+2\right)n_{arr}}+2\sqrt{\frac{n_{arr}}{N+2}}\right)$$

where *N* corresponds to the number of electrodes (16 in our case) and *n_arr_* corresponds to number of training electrode arrays (2918 in our case). The number of neurons in layer 2 (*n_l_*_2_) is given by equation (8)

(8) $$n_{/2}=\text{floor}\left(N\sqrt{\frac{n_{arr}}{N+2}}\right).$$

The input of the NN is composed of neurons accepting selected figures of merit, while the output consists of a regression layer. This layer compares a flattened vector of the 3D coordinates of electrode positions corresponding to the respective input feature values to electrode positions computed by the NN based on input values. For the 16 electrodes in three dimensions used in this work, a regression layer with 48 neurons was needed. The entire network scheme is described in Table 3.

**Table 3:** Overview of layers for an electrode position optimization NN.

#	Layer type	Layer information
1	input layer	3-element vector
2	FC layer	254 neurons
3	ReLU layer	activation layer
4	FC layer	203 neurons
5	ReLU layer	activation layer
6	FC layer	48 neurons
7	regression layer	determine positions

### Thrombus detection

The most fundamental question of whether any thrombi are deposited in the oxygenator was approached by a classification NN. Training data were generated using simulations with the optimal electrode array with the respective optimized I-M pattern. A target grid with 3,971 targets, each 1 cm in diameter, was used for inhomogeneous simulations. Only one target was used for each inhomogeneous simulation to test the basic scenario with one deposited thrombus. Target conductivity was randomly varied around the standard value *σ_target_* based on a normal distribution of $N\left(0,\frac{\sigma_{t\arg et}}{10}\right)$ . A second group of training data consisted of noise-only simulations as a negative control. A normal random noise with a distribution of $N\left(0,\frac{\sigma_{back}}{100}\right)$ was added to the homogeneous background distribution *σ_back_*. Simulated voltage measurements for both groups were augmented with 15 dB SNR noise. Overall, 3,971 inhomogeneous simulations and 3,971 noise simulations were created for training with ∆V*_r_* data as the NN input. In practice, when a new oxygenator is deployed, the first voltage measurement is recorded as the homogeneous case and further voltage measurements are treated as inhomogeneous. The observed changes are hypothesized to be attributable to variations in conductivity resulting from clot accumulation. Input data was randomly partitioned into three sets to form training (80 %), validation (10 %) and test datasets (10 %) respectively.

The NN has an input layer accepting measured voltages in the form of a 208-element vector. It further consists of two FC inner layers paired with a ReLU activation function. The output of the NN is composed of a FC layer with two neurons and a softmax layer. This arrangement enables classification into two groups – thrombi present or thrombi missing. The entire network scheme is described in Table 4.

**Table 4:** Overview of layers for a thrombus detection NN.

#	Layer type	Layer information
1	input layer	208-element vector
2	FC layer	200 neurons
3	ReLU layer	activation layer
4	FC layer	100 neurons
5	ReLU layer	activation layer
6	FC layer	2 neurons
7	softmax layer	to probabilities
8	classification layer	more probable class

### Ethical approval

The conducted research is not related to either human or animal use.

## Results and discussion

### FEM modelling

**Figure 6:**
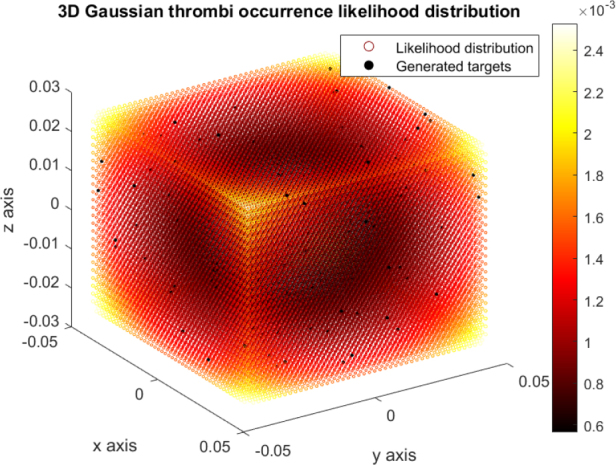
3D thrombus occurrence likelihood distribution with a sample set of generated thrombi.

The internal flow field model indicates the highest likelihood of thrombus occurrence in the corners of the oxygenator, where flow stagnates the most. A cumulative distribution function (CDF) was created for the possibility of a pseudo-random selection of thrombi locations based on a given CDF percentile. The likelihood distribution is symmetrical with respect to the center of the oxygenator with ellipsoidal isolines, therefore more voxels with the same respective thrombi likelihood exist for one concrete CDF value. The thrombi location selection is therefore complemented by a random choice from these options. An example of a generated set of thrombi is depicted in Figure 6.

### Optimized electrode array

Pearson correlation was performed over all designed figures of merit to pinpoint redundant and useful sources of information. It also revealed several unexpected findings, such as a significant divergence between some figures of merit based on ∆V*_r_* and *J* . Mean ∆V*_r_* was only weakly correlated to median ∆V*_r_* (r = 8.84 × 10^−2^), whereas mean *J* was strongly correlated to median *J* (r = 0.93). Mean ∆V*_r_* was strongly correlated to max ∆V*_r_* (r = 0.99), whereas mean *J* was weakly correlated to max *J* (r = −0.11). Two interesting results regarding *J* were observed as well. Rank of the *J* was strongly negatively correlated to most of the figures of merit tied to the *J* (e.g. r = −0.61 for mean *J* and r = −0.65 for min *J* ). Feature $\frac{medianJ}{\min J}$ was strongly negatively correlated to max *J* (r = −0.87). Three uncorrelated figures of merit regarding homogeneity described in Table 5 were chosen as the NN input.

**Table 5:** Figures of merit chosen for position optimization NN training.

Feature	Explanation
$\frac{\text{median}\;\text{J}}{\text{min}\;\text{J}}$	*J* homogeneity
$\frac{\text{max}\sigma_{k}}{\text{min}\sigma_{k}}$	*J* condition number
$\frac{\text{median}\Delta \text{V}}{\min\Delta \text{V}}$	∆V*_r_* measurement homogeneity

**Figure 7:**
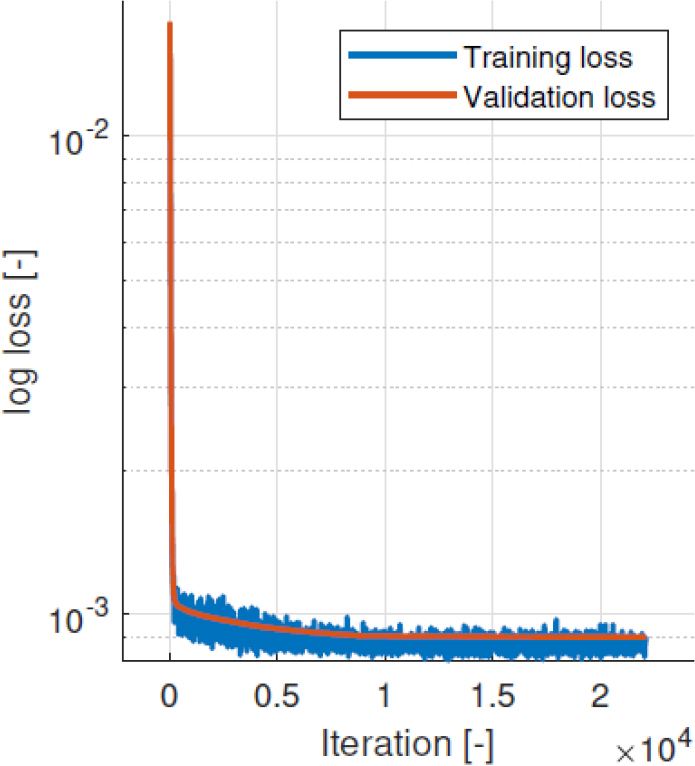
Time course of loss for electrode array optimization NN training.

The NN training was successful, with both validation and training losses steadily declining as depicted in Figure 7. In order to obtain the optimized electrode positions, scaled input feature values theoretically corresponding to an optimal electrode array were used as an input vector to the NN. Afterwards, a forward pass was carried out and optimal electrode array coordinates were read from NN output. Optimal feature values were chosen as [1, 1, 1] to maximize homogeneity. The output coordinates from the NN output were assigned to nearest plausible positions.

**Figure 8:**
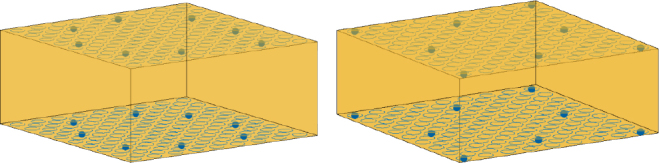
Electrode array for optimal feature values (left) and adjusted electrode array (right).

**Figure 9:**
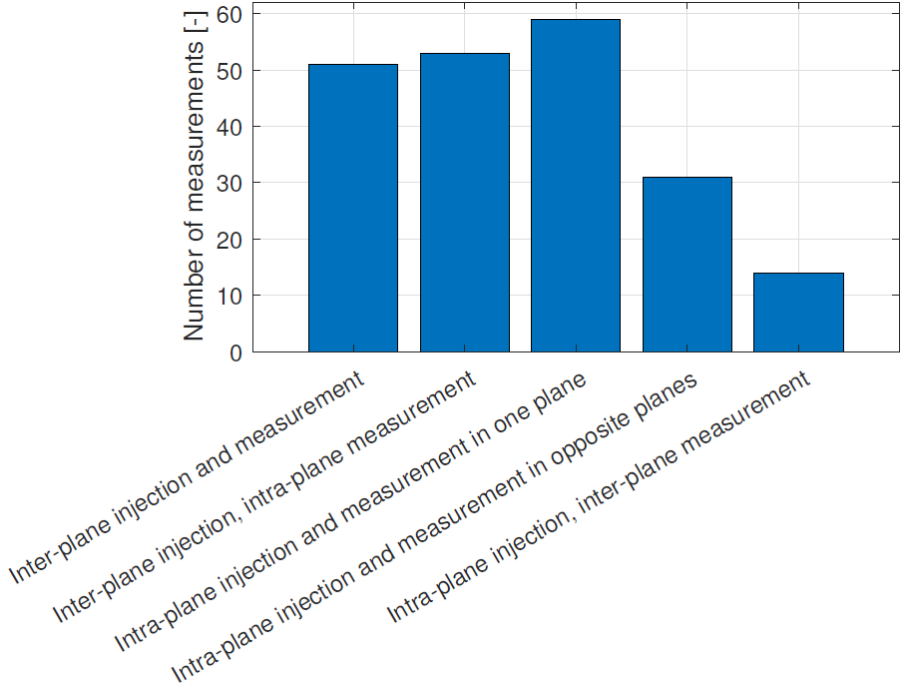
Information about spatial arrangement of used pairs of the adjusted electrode array.

The electrodes are homogeneously placed. However, they do not cover well the areas with the highest probability of thrombus occurrence near edges and corners. As mentioned before, it is a reasonable constraint to assume electrode positioning might be done symmetrically, as the internal structure of the oxygenator also is. Integrating this sort of prior into the training process of the neural network is beyond the scope of this work, and could lead to confounding factors in our results. Therefore, we manually adjusted for this heuristic based on the algorithm output as can be seen in Figure 8. The respective I-M pattern contains 56 different injecting pairs and a total of 208 measurements. Half of the chosen measurements stem from sensitivity vector parallelotope maximization, which brings the best possible homogeneous coverage of all finite elements. The other half comes from the maximization of L1- and L2-norms of the sensitivity vectors. Summarizing information about used pairs is depicted in Figure 9. The biggest proportion of measurements is conducted with both injecting and measuring pairs in the same plane. These measurements are highly sensitive mostly to the thrombi adherent to the walls of the oxygenator. Overall, selected measurements bring amplification of useful signal and mitigate noise influence.

### Thrombus detection

**Figure 10:**
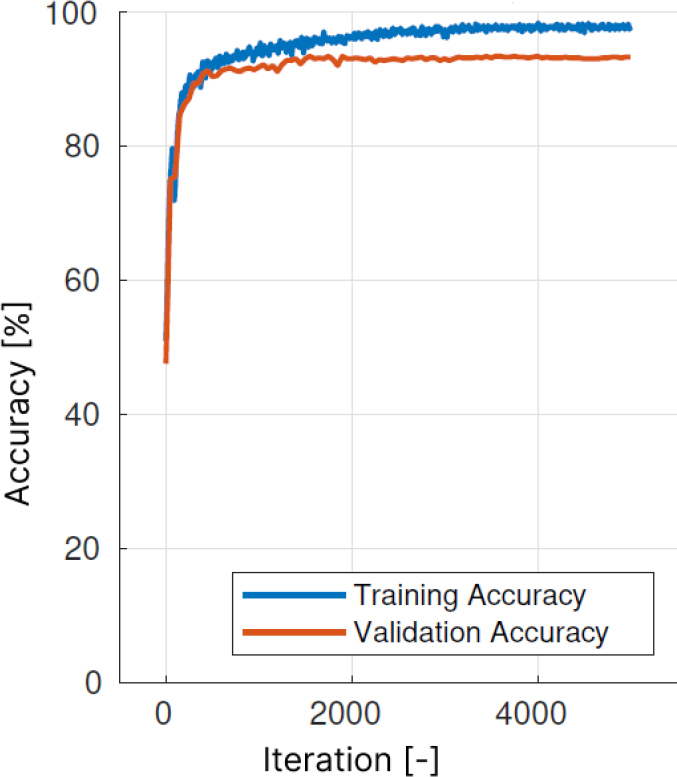
Time course of accuracy for thrombus detection NN training.

**Figure 11:**
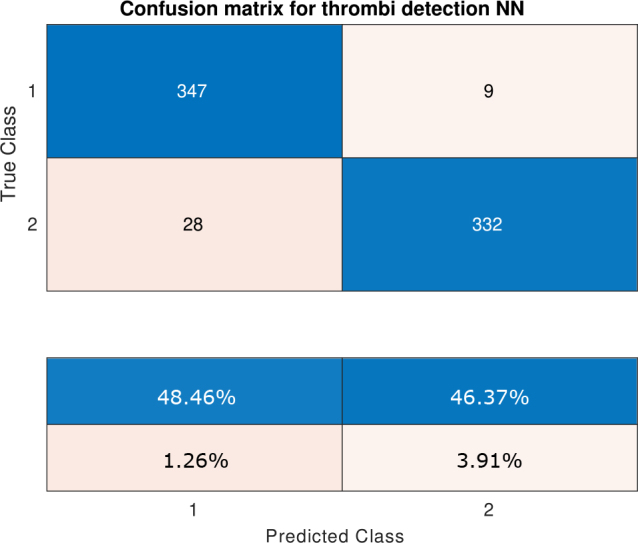
Confusion matrix for the thrombus-detection NN. Class #1 corresponds to thrombi not present and class #2 corresponds to thrombi present.

The classifier training was successful, as evidenced by the consistent increase in both validation and training accuracy in Figure 10. The accuracy on training and testing dataset proved to be similar as well, further proving no overfitting happened with respect to the dataset. A sensitivity over 92 %, a precision over 97 % and a F1-score over 94 % was achieved for test data. The confusion matrix in Figure 11 shows 1.3 % of false positive cases, where thrombus was detected in the oxygenator when it was not present. This scenario would require additional checks by staff, but would not put the patient’s life at risk. It is important to minimise the number of false positives in practical application, which were observed in 1.3 % of cases.

## Conclusions

This study demonstrates, *in silico*, that multi-electrode bioimpedance measurements combined with shallow neural networks can reliably identify thrombotic inclusions inside a commercial ECMO oxygenator model. Through a sensitivity-guided measurement design and data-driven optimization of electrode placement and injection–measurement schemes, we achieved a high level of accuracy, with an F1-score greater than 94%, in differentiating thrombus-affected from nominal operating conditions.

The study acknowledges that relying exclusively on FEM simulations represents a limitation, as the absence of experimental validation can raise questions about clinical applicability. Nevertheless, several factors specific to the oxygenator context strengthen the robustness of the simulations: a static and closed measurement volume; distinct electrical properties of blood, thrombi, and structural materials; slow thrombus formation dynamics; the availability of a clear baseline measurement after device priming. These characteristics substantially mitigate typical confounding factors encountered in more complex bioimpedance scenarios, allowing controlled exploration of methodological strengths and limitations prior to costly experimental setups.

Moreover, the utilization of simulated data, despite being deterministic in nature, provides significant practical benefits. Gathering extensive labeled experimental data from actual clotting events would necessitate hundreds of trials, a process currently impractical due to logistical and ethical constraints. By augmenting simulations with realistic physiological variability, such as randomized electrode positions, stochastic thrombus placements, conductivity variability, and added measurement noise, we established a diverse training dataset. This strategy aligns with established practices in similarly critical domains [17, 18] where synthetic-to-real transfer learning is commonly employed to bootstrap model development and direct experimental investigations.

The next step involves conducting an experimental validation campaign using a blood-perfused oxygenator mock loop. Experimental data obtained from this setup will validate and quantify the accuracy of our simulation models, refine classifiers through domain adaptation, and guide subsequent methodological enhancements. Further studies will explore more realistic thrombus morphologies informed by fluid dynamics, enhance classifier granularity to localize and size thrombi accurately, and investigate the transferability of the optimization methods to broader clinical and perfusion monitoring applications.

In summary, this study serves as a foundational step towards eventual clinical adoption (rather than an endpoint in itself) through a simulation-based approach within a broader translational strategy encompassing ECMO oxygenator as well as analogous multi-channel bioimpedance applications.

## References

[1] Brodie D, Slutsky AS, Combes A (2019). Extracorporeal Life Support for Adults With Respiratory Failure and Related Indications: A Review. JAMA..

[2] Teijeiro-Paradis R, Gannon WD, Fan E (2022). Complications Associated With Venovenous Extracorporeal Membrane Oxygenation—What Can Go Wrong?. Critical Care Medicine.

[3] Iannattone PA, Yang SS, Koolian M, Wong EG, Lipes J (2022). Incidence of Venous Thromboembolism in Adults Receiving Extracorporeal Membrane Oxygenation: A Systematic Review. ASAIO Journal.

[4] Dornia C, Philipp A, Bauer S, Lubnow M, Müller T, Lehle K, Schmid C, Müller-Wille R, Wiggermann P, Stroszczynski C, Schreyer AG (2014). Analysis of Thrombotic Deposits in Extracorporeal Membrane Oxygenators by Multidetector Computed Tomography. ASAIO Journal.

[5] Türkmen M, Lauwigi T, Fechter T, Gries F, Fischbach A, Gries T, Rossaint R, Bleilevens C, Winnersbach P (2023). Bioimpedance Analysis as Early Predictor for Clot Formation Inside a Blood-Perfused Test Chamber: Proof of Concept Using an In Vitro Test-Circuit. Biosensors.

[6] Gelfan S, Quigley JP (1930). Conductivity of blood during coagulation. American Journal of Physiology-Legacy Content.

[7] Noshiro M, Nebuya S, Fujimaki A, Smallwood R, Brown B (2007). Frequency characteristics of the electrical conductivity in normal and coagulated blood. IFMBE Proceedings.

[8] Sapkota A, Fuse T, Seki M, Maruyama O, Sugawara M, Takei M (2015). Application of electrical resistance tomography for thrombus visualization in blood. Flow Measurement and Instrumentation.

[9] Chen H, Yao J, Yang L, Liu K, Chen B, Li J, Takei M (2021). Development of a Portable Electrical Impedance Tomography Device for Online Thrombus Detection in Extracorporeal-Circulation Equipment. IEEE Sensors Journal.

[10] Hathcock JJ (2006). Flow Effects on Coagulation and Thrombosis. Arteriosclerosis, Thrombosis, and Vascular Biology.

[11] Istuk N, Gioia AL, Benchakroun H, Lowery A, Mc-Dermott B, O’Halloran M (2022). Relationship Between the Conductivity of Human Blood and Blood Counts. IEEE Journal of Electromagnetics, RF and Microwaves in Medicine and Biology.

[12] Baumgartner C, Hasgall PA, Di Gennaro F, Neufeld E, Lloyd B, Gosselin MC, Payne D, Klingenböck A, Kuster N (2024). IT’IS Database for Thermal and Electromagnetic Parameters of Biological Tissues, Version 4.2.

[13] Adler A, Lionheart WRB (2006). Uses and abuses of EIDORS: an extensible software base for EIT. Physiological Measurement.

[14] Onsager C, Wang C, Costakis C, Aygen C, Lang L, Lee S van der, Grayson MA (2021). Sensitivity Analysis for Optimizing Electrical Impedance Tomography Protocols.

[15] Korte J, Lauwigi T, Herzog L, Theißen A, Suchorski K, Strudthoff LJ, Focke J, Jansen SV, Gries T, Rossaint R, Bleilevens C, Winnersbach P (2024). Prediction of Thrombus Formation within an Oxygenator via Bioimpedance Analysis. Biosensors.

[16] Smyl D, Liu D (2020). Optimizing Electrode Positions in 2-D Electrical Impedance Tomography Using Deep Learning. IEEE Transactions on Instrumentation and Measurement.

[17] Silva D, Leonhardt S, Antink CH (2021). Copula-Based Data Augmentation on a Deep Learning Architecture for Cardiac Sensor Fusion. IEEE Journal of Biomedical and Health Informatics.

[18] Rixen J, Blass N, Lyra S, Leonhardt S (2023). Comparison of Machine Learning Classifiers for the Detection of Breast Cancer in an Electrical Impedance Tomography Setup. Algorithms.

